# Destruxin B Isolated from Entomopathogenic Fungus *Metarhizium anisopliae* Induces Apoptosis via a Bcl-2 Family-Dependent Mitochondrial Pathway in Human Nonsmall Cell Lung Cancer Cells

**DOI:** 10.1155/2013/548929

**Published:** 2013-09-24

**Authors:** Chun-Chi Wu, Tzu-Hsiu Chen, Bing-Lan Liu, Li-Chen Wu, Yung-Ching Chen, Yew-Min Tzeng, Shih-Lan Hsu

**Affiliations:** ^1^Institute of Medicine, Chung Shan Medical University, Taichung, Taiwan; ^2^Department of Medical Research, Chung Shan Medical University Hospital, Taichung, Taiwan; ^3^Department of Health and Nutrition, Chia Nan University of Pharmacy & Science, Tainan, Taiwan; ^4^Department of Applied Chemistry, Chaoyang University of Technology, Taichung, Taiwan; ^5^Department of Applied Chemistry, National Chi Nan University, Puli, Nantou, Taiwan; ^6^Department of Education and Research, Taichung Veterans General Hospital, Taichung, Taiwan

## Abstract

Destruxin B, isolated from entomopathogenic fungus *Metarhizium anisopliae*, is one of the cyclodepsipeptides with insecticidal and anticancer activities. In this study, destruxin B was extracted and purified by ion-exchange chromatography, silica gel chromatography, and semipreparative high-performance liquid chromatography. The potential anticancer effects and molecular mechanisms of destruxin B in human nonsmall cell lung cancer cell lines were characterized. Our results showed that destruxin B induced apoptotic cell death in A549 cells. This event was accompanied by the activation of caspase-2, -3, and -9. Moreover, destruxin B increased the expression level of proapoptotic molecule, PUMA, while decreased antiapoptotic molecule Mcl-1. Additionally, the translocation of Bax from cytosol to mitochondrial membrane was observed upon destruxin B treatment. Knockdown of Bax by shRNA effectively attenuated destruxin-B-triggered apoptosis in A549 cells. Interestingly, similar toxic effects and underlying mechanisms including caspase activation, upregulation of PUMA, and downregulation of Mcl-1 were also observed in a p53-null lung cancer H1299 cell line upon destruxin B treatment. Taken together, our findings suggest that destruxin-B-induced apoptosis in human nonsmall cell lung cancer cells is via a Bcl-2 family-dependent mitochondrial pathway.

## 1. Introduction

Lung cancer is the leading cause of death for both men and women in many countries, including Taiwan, which exhibited the highest rate of increase in lung cancer mortality in recent years [1, 2]. The five-year survival rate of lung cancer patient is only 13–25% [3]. Although target therapy emerged for treatment of lung cancer patients in the past decade [4], there is still a large amount of patients who are uncured. It appears that the drugs with greater efficacy than existing treatments are urgently required for these patients.

Growing evidence demonstrates that apoptosis is involved in many biological events including physiological homeostasis and embryogenesis [5] and is also an important determinant of the response of cancers to chemo-, radiation, or target-therapy [6, 7]; compounds that induce these events may provide potent anticancer effects for cancer treatment. The regulatory mechanisms of apoptosis are quite complicated networks. Two major types of apoptosis pathways are mentioned: one is the intrinsic pathway which is mediated by the regulators located in the mitochondrial [8] and the other is the extrinsic pathway which is regulated by the binding of the membrane receptor to the death ligand such as TNF (tumor necrosis factor)/TNFR and Fas/FasL [9]. The apoptotic process is mainly executed by a class of cysteine proteases known as caspases [10]. Activation of different caspases usually presents for various upstream apoptotic pathways. For example, the activation of caspase-8 and -10 usually stands for the extrinsic/death receptor-related pathway; caspase-2 and caspase-9 are usually activated by mitochondrial pathway, while caspase-12 is activated by endoplasmic reticulum pathway [10]. In addition, the activation of caspase-3, -6, or -7 is usually considered as a common executor of apoptosis by directly cleaving cellular proteins when activated [11].

Several proteins are known to be involved in the regulation of apoptosis; among them Bcl-2 family molecules contribute to the mitochondrial-mediated apoptotic pathway. Bcl-2 is originally found in a translocation chromosomal fragment in B-cell lymphoma and is proved as a protooncogene [12]. The members of Bcl-2 family all contain a BH (Bcl-2 homology) domain structure and are divided into two major groups: one composed of antiapoptotic molecules, including Bcl-2, Bcl-xL, Bcl-w, Mcl-1, Boo/Diva, and A1/Bfl-1 [13] and the other comprises proapoptotic molecules, such as Bax, Bak, Bad, Bim, Bik, PUMA, and NOXA [14]. Under normal conditions, the Bcl-2 or Bcl-xL complexes with Bax or Bak to form homo- or hetero dimer, and such interaction will neutralize proapoptotic molecules to prevent cell death. Under apoptotic stimulation, however, it will change the ratios between these two groups, leading to the formation of Bax/Bax or Bax/Bak complexes followed by perforation through the membrane of mitochondria. The opened pores eventually cause the release of cytochrome c from mitochondria to the cytosol and form apoptosis complex with Apaf-1 (apoptotic protease activation factor-1), dATP (deoxyadenosine triphosphate), and procaspase-9 to induce the caspase-9 and caspase-3 activation and irreversible apoptosis [15].

It is well known that certain compounds isolated from bacteria or fungus carry characteristic biological effects, which have been used as traditional medicines [16, 17], and several of these compounds have shown an antitumoral activity [18–20]. One group of such biological active compounds is depsipeptides, which are the major secondary metabolites of fungi. The depsipeptides are biooligomers formed by esterification between hydroxyl groups and amino acids. Destruxin B is a cyclic depsipeptide isolated from entomopathogenic fungus in 1961. Until recently, there were 39 destruxins reported [21]. It has been reported that cyclic depsipeptides exhibited strong antitumor activity in both *in vitro* and *in vivo* experiments and evenly entered the clinical trial [22]. Previous reports mentioned that destruxin B has strong anti-insect activity [23], inhibits hepatitis B surface antigen expression, and induces cell death in hepatocellular carcinoma cells [24] and leukemic cells [25]. The effects of destruxin B on lung cancer cells and its molecular mechanisms, however, have not been examined yet. In this study, the antiproliferative, cytotoxic effects and underlying mechanisms of destruxin B (isolated and purified from fermentation broth of *Metarhizium anisopliae*) on lung cancer cell lines were explored. Our observations indicate that destruxin B induces a Bcl-2 family-dependent mitochondrial apoptotic cell death in human nonsmall cell lung cancer A549 and H1299 cell lines.

## 2. Material and Methods

### 2.1. Reagents

The destruxin B (MW 593) was dissolved in ethanol to a stock solution at concentration of 20 mM for the following experiments. Anti-Bax, Bcl-2, Bcl-xL, Bak, PUMA, Mcl-1, and *β*-actin were purchased from Santa Cruz Biotechnology (Santa Cruz, CA, USA) or Calbiochem Chemical Company (CN Biosciences, Notts, UK). Anti-mouse and anti-rabbit IgG peroxidase-conjugated secondary antibodies were purchased from Amersham (Buckinghamshire, UK). Caspase activity assay kits, including the fluorogenic substrates of caspase-2 (VDVAD-AFC), caspase-3 (DEVD-AFC), and caspase-9 (LEHD-AFC), were purchased from R&D systems (Minneapolis, MN, USA). The caspase-2 inhibitor (z-VDVAD-FMK) was purchased from Santa Cruz Biotechnology (Santa Cruz, CA, USA). The caspase-3 inhibitor (Z-DEVD-FMK) and caspase-9 inhibitor (Z-LEHD-FMK) were purchased from Kamiya (Seattle, WA, USA). 

### 2.2. Isolation and Purification of Destruxin B

The destruxin B used in this study was extracted and purified from the submerged cultivation of fungus *Metarhizium anisopliae* F061 var. Anisopliae according to the processes described elsewhere [26, 27]. Briefly, *Metarhizium anisopliae* was cultivated in 3% maltose containing 0.5% bactopeptone liquid medium, at 28°C. After 14 days, the culture medium was collected and extracted three times with equal volume of methylene dichloride (CH_2_Cl_2_). The organic layer was collected and concentrated and further separated by two-step ion-exchange chromatography. Combination of cationic (Amberlite TRA-400 CP column) and anionic (Amberlite IR 120 Plus) exchange provided the step to removing charged impurities existing in the mixture, and then the eluent was collected and subjected to flash silica chromatography (Merck K-60 silica gel column) as a prepurification step. The column was eluted with a methylene dichloride-methanol mixture. Further purification was done by loading the sample into a semipreparative RP-C18 high-performance liquid chromatographic (HPLC) column. The which elution delivered a single peak was collected and concentrated. This peak represented destruxin B (retention time = 17.6 min). The purity of destruxin B in the sample was proved >95% in our early report [28].

### 2.3. Cell Culture and Viability Assay

The human lung cancer A549 and H1299 cells were obtained from CCRC (Culture Collection and Research Center, Hsinchu, Taiwan). The A549-*sh*Bax cells were established by transfection of pSuper-*sh*Bax followed by antibiotics selection [29]. A549, A549-*sh*Bax, and H1299 cells were cultured in RPMI (GIBCO/BRL, MD, USA). All cultured media were supplemented with 10% fetal bovine serum, 2 mM glutamine, and antibiotics (100 U/mL penicillin and 100 *μ*g/mL streptomycin) at 37°C in a humidified atmosphere of 5% CO_2_. The medium was changed every 48 h. For cell viability assay, cells were seeded in 12-well plates at a density of 1 × 10^5^ cells per well. After 24 h, cells were treated with various concentrations of destruxin B for the indicated time points. After treatment, the number of viable cells was determined by trypan-blue dye exclusion method. 

### 2.4. Cell Cycle Analysis

Cells were seeded as a number of 2 × 10^5^ cells/well onto 6-well plates. After 24 h incubation, different concentrations of destruxin B (0, 10, 20, and 30 *μ*M) were added for another 48 h. The cells were then collected and washed with cold PBS twice followed by centrifugation at 1200 rpm at 4°C. Cell pellet was suspended with 1 mL cold ethanol (70%) and then was incubated with buffer containing propidium iodide (40 *μ*g/mL) and RNase A (100 *μ*g/mL) for 30 min under dark condition. The prepared cell samples were subjected to flow cytometry (FACScan) and analyzed by ModFIT LT software.

### 2.5. Caspase Activity Assay

Cell lysates obtained from destruxin-B-treated or -untreated cells were tested for caspase-2, -3, -9 activities by addition of a caspase-specific peptide substrate conjugated with the fluorescent reporter molecule 7-amino-4-trifluoromethyl coumarin (R&D Systems, Minneapolis, USA). The cleavage of the peptide by the caspase releases the fluorochrome that when excited by light at 400 nm emits fluorescence at 505 nm. The level of caspase enzymatic activity in the cell lysate is directly proportional to the fluorescence signal detected using a fluorescent microplate reader (Fluoroskan Ascent; Labsystems, Finland).

### 2.6. Protein Preparation and Immunoblotting

Cells were cultured without or with destruxin B at various time points. After treatment, both adherent and floating cells were harvested, washed twice with ice-cold PBS, and decomposed in ice-cold modified RIPA buffer (50 mM Tris-HCl (pH 7.4), 150 mM NaCl, 1% Triton X-100, 0.25% Na-deoxycholate, 5 mM ethylenediamine-tetra-acetic acid (pH 8.0), 1 mM ethylene glycol-bis(2-aminoethylether)-N′,N′N,N′-tetra-acetic acid (pH 8.0), 1 mM dithiothreitol, 0.2 mM phenylmethylsulfonylfluoride, 5 *μ*g/mL leupeptin, 5 *μ*g/mL aprotinin, and 1 mM sodium orthovanadate). After 30 min incubation on ice, cells were centrifuged at 100,000 g for 30 min at 4°C, and supernatants were collected. Protein concentration was determined using the Bradford method. For western blot analysis, equal amounts of total proteins were loaded onto SDS-polyacrylamide gels followed by electrophoretically transferring onto a PVDF membrane (Millipore, MA, USA). Membranes were incubated with specific primary antibodies overnight. After exposure to horseradish peroxidase-conjugated secondary antibody for 1 h, proteins were visualized using an enhanced chemiluminescence detection kit (ECL Kits; Amersham Life Science, NJ, USA).

### 2.7. Indirect Immunofluorescence

A549 cells were trypsinized and seeded onto coverslips. After reaching 75% confluence, cells were treated with destruxin B for 24 h. Cells were washed with PBS twice and stained with MitoTracker for 15–30 min. After treatment, cells were washed with PBS and fixed with ethanol at −20°C for 20 min, incubated with Bax antibody at room temperature for 4 h, and then washed three times with PBS and incubated with goat-anti-rabbit-FITC (Jackson, PA, USA) and 1 mg/mL DAPI (Sigma, MO, USA) for another 1 h. After washing with PBS containing 0.1% Tween 20, the samples were mounted. The fluorescence images of the stained cells were analyzed using a Zeiss fluorescence microscope.

### 2.8. RNA Extraction and Quantitative RT-PCR

Total cellular RNA was extracted by RNA-Bee RNA isolation kit (TEL-TEST, Friendswood, TX) according to the manufacturer's instructions. One microgram of total RNA was reverse-transcribed using Advantage RT for PCR Kit (Clontech, CA, USA) at 42°C for 1 hr. The PCR primers were listed as follows for Mcl-1 forward primer: AAAGAGGCTGGGATGGGTTT and reverse primer: CAAAAGCCAGCAGCACATTC; for PUMA forward primer: CCTGGAGGGTCCTGTACAATCT and reverse primer: GCACCTAATTGGGCTCCATCT; for *β*-actin forward primer: GCAAATGCTTCTAGGCGGACTAT and reverse primer: TGCGCAAGTTAGGTTTTGTCA. The mRNA levels were determined by real-time PCR with ABI PRISM 7900 Sequence Detector system according to the manufacturer's instructions. *β*-Actin was used as endogenous control. PCR reaction mixture contained the SYBR PCR master mix (Applied Biosystems), cDNA, and the primers. Relative gene expression level (the amount of target, normalized to endogenous control gene) was calculated using the comparative Ct method formula *E* ^−ΔΔCt^.

### 2.9. Statistical Analysis

Figures were obtained from at least 3 independent experiments with similar patterns. All data are presented as mean ± S.D. of 9 replicates from 3 separate experiments. Statistic differences were evaluated using Student's *t*-test, with *P < 0.05, **P < 0.01, and ***P < 0.001 considered significant, or by the calculation and grouping using SAS program.

## 3. Results

### 3.1. Preparation of Destruxin B

**Figure 1:**
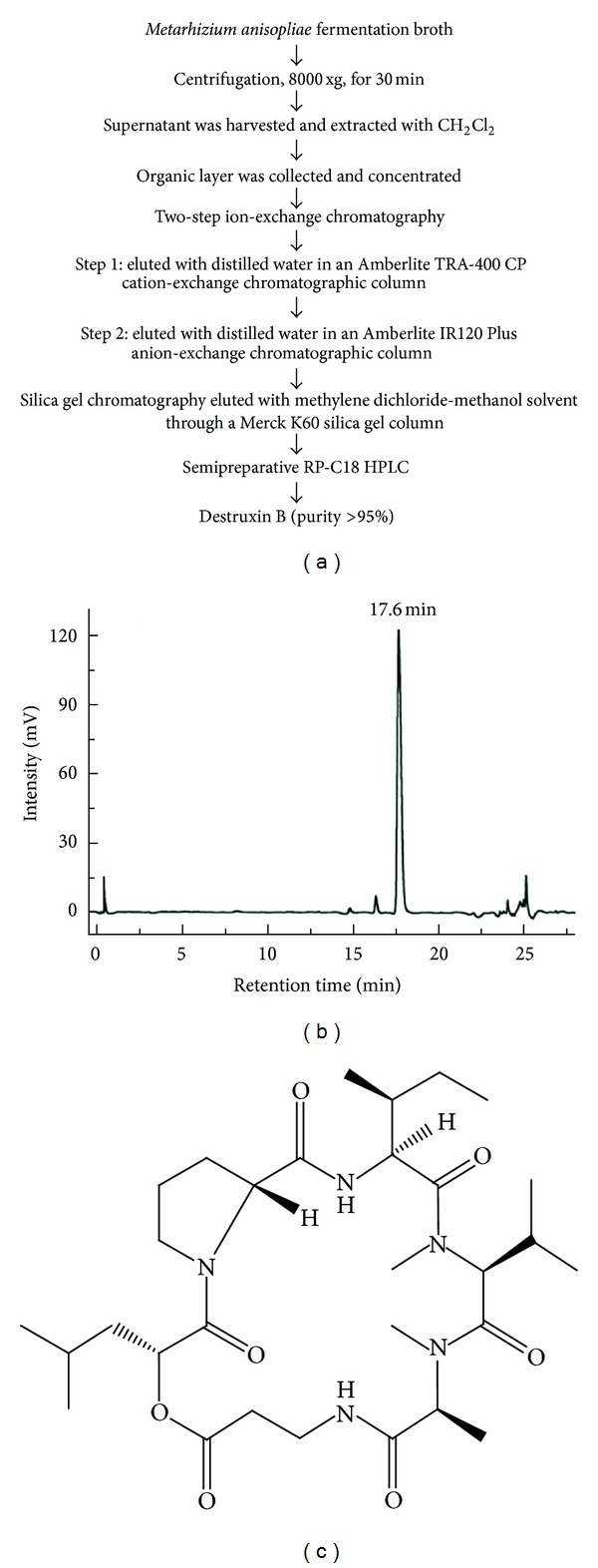
Isolation and purification of destruxin B from *Metarhizium anisopliae* fermentation broth. (a) Flowchart of the extraction and purification procedure. (b) HPLC chromatographic spectrum of purified destruxin B. *Metarhizium anisopliae* fermentation broth was centrifuged and filtered. The filtrate was extracted with CH_2_Cl_2_, and then the destruxin B was extracted and purified by ion-exchange chromatography, silica gel chromatography, and HPLC. (c) The chemical structure of destruxin B.

Destruxin was isolated, purified, and identified from the fermentation broth of submerged cultivation of fungus *Metarhizium anisopliae* F061 var. Anisopliae by CH_2_Cl_2_ extraction, ion-exchange chromatography, silica gel chromatography, and HPLC quantification.  Figure 1(a) presents a schematic diagram of extraction and isolation procedures. An HPLC fingerprint chromatogram was performed for the quantification and quality control of the isolated samples (Figure 1(b)), which showed only one major peak (retention time = 17.6 min). The peak was identified as destruxin B. The purity of destruxin B in the sample was proved >95% in our early report [28]. The chemical structure of destruxin B is presented in Figure 1(c).

### 3.2. Destruxin B Induces a Caspase-Dependent Apoptosis

**Figure 2:**
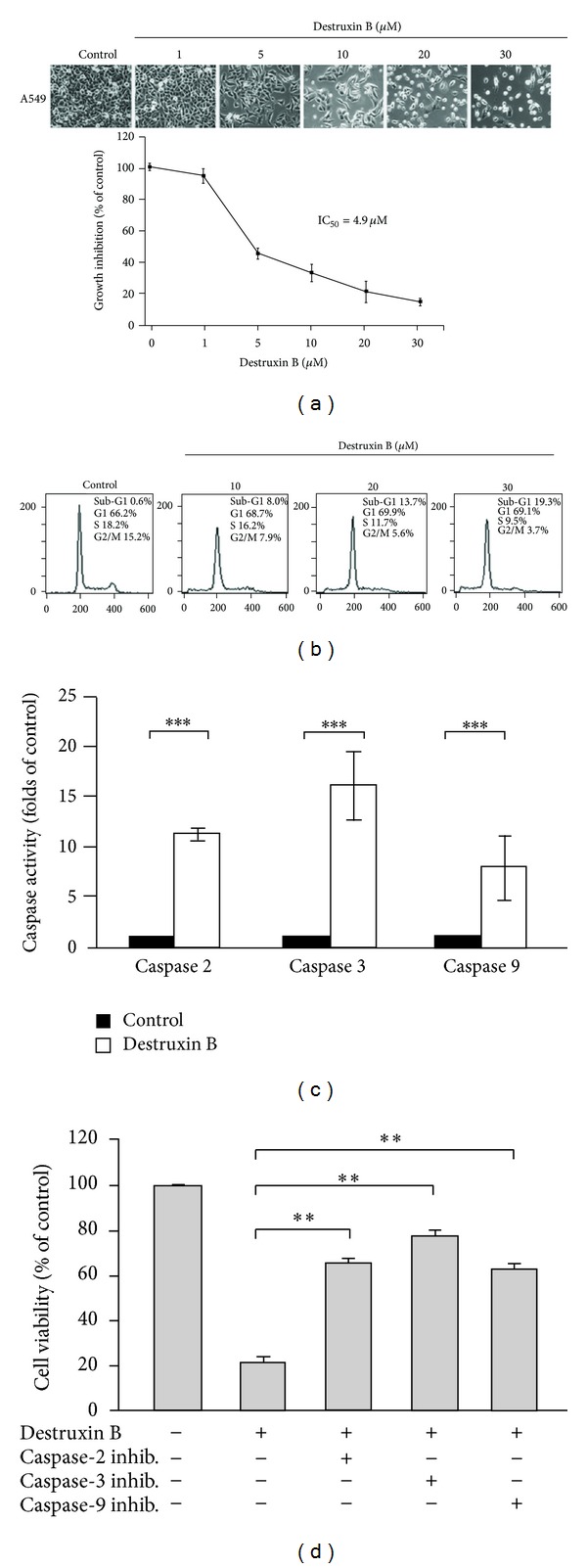
Destruxin B induces a caspase-dependent cell death. (a) Antiproliferative effect of destruxin B. Human nonsmall cell lung cancer A549 cells were administrated with different concentrations of destruxin B (0, 1, 5, 10, 20, and 30 *μ*M) for 48 h. The cellular morphology was observed under microscope and the cell numbers were then counted by trypan-blue dye exclusion method using hemocytometer. (b) Sub-G1 population is increased by destruxin administration. A549 cells were treated with different concentrations of destruxin B (0, 10, 20, and 30 *μ*M) for 48 h. Cells were then fixed with ethanol and subjected to flow cytometry assay. (c) Destruxin B induces caspase activation. A549 cells were treated with 20 *μ*M destruxin B for 48 h. Cell lysates were harvested and the indicated caspase activity was measured as described in Section 2. (d) Inhibition of caspase activity blocks destruxin-B-triggered cell death. A549 cells were pretreated with indicated caspase inhibitors for 1 h and then treated with 20 *μ*M destruxin B for another 48 h. The cell viability was determined by trypan-blue dye exclusion methods (**P < 0.01 versus control).

To examine the growth inhibitory effect of destruxin B, different doses of destruxin B (0, 1, 5, 10, 20, and 30 *μ*M) were added into lung adenocarcinoma A549 cells. As shown in Figure 2(a), destruxin B induced a concentration-dependent antiproliferative effect on A549 cells, with IC_50_ value at 4.9 *μ*M. Treatment with low concentrations of destruxin B resulted in growth inhibition but not cell death. However, destruxin-B-induced cytotoxicity occurred at concentration up to 10 *μ*M (Figure 2(a)). Data from cell flow cytometric analysis showed that destruxin B induced a sub-G1 population accumulation at 10~30 *μ*M destruxin B administration (Figure 2(b)), indicating that destruxin B triggered the apoptotic cell death of A549 cells. To address whether destruxin-B-induced apoptosis was through a caspase activation pathway, fluorogenic peptide substrates were used to examine the specific caspase activity. As depicted in Figure 2(c), caspase-2, -3, and -9 were significantly activated after 48 h destruxin incubation, while caspase-8 and caspase-12 were not activated upon destruxin B treatment (data not shown). To confirm the role of caspase activation in destruxin-B-mediated apoptosis, A549 cells were pretreated with caspase inhibitors prior to destruxin B. In Figure 2(d), the inhibitors of caspase-2, caspase-3, and caspase-9 drastically attenuated destruxin-B-induced cell death, whereas the inhibitors of caspase-8 and caspase-12 did not affect the destruxin-induced cell death (data not shown). These observations indicate that activation of mitochondria-dependent caspase cascade might play an important role in destruxin-B-induced apoptotic cell death in A549 cells.

### 3.3. Bcl-2 Family Molecules Contribute to Destruxin-B-Mediated Apoptosis

**Figure 3:**
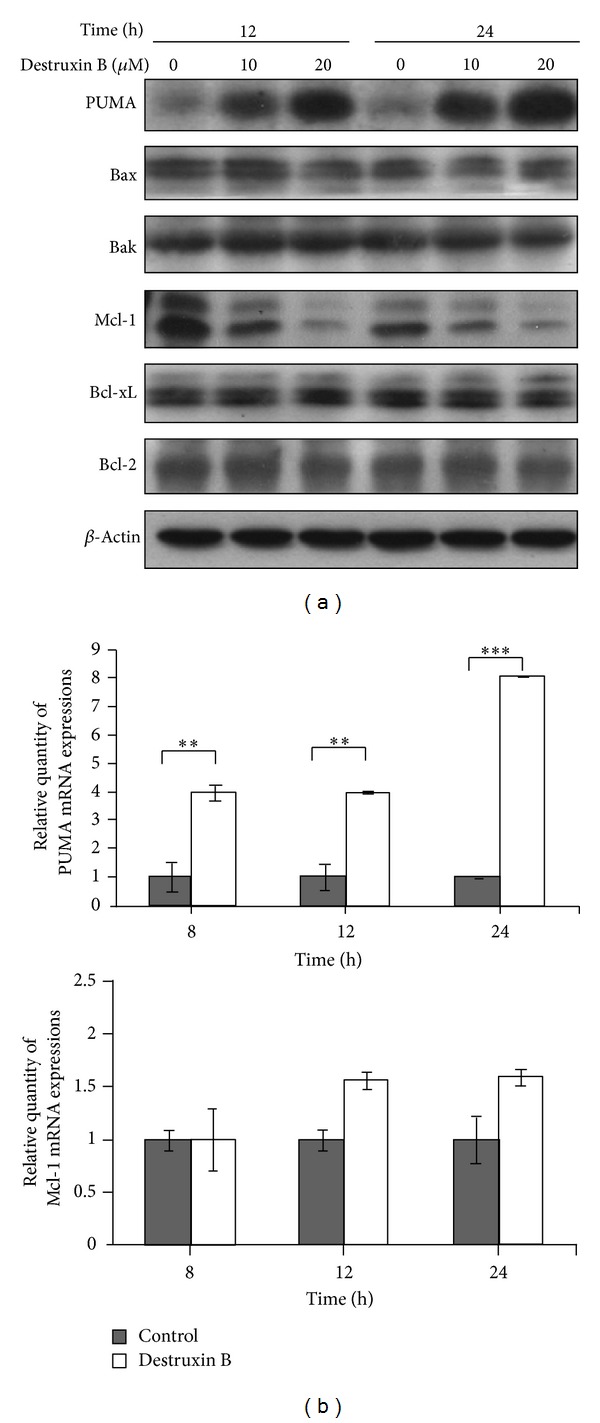
Regulation of Mcl-1 and PUMA by destruxin B. (a) Regulation of Bcl-2 family proteins by destruxin. A549 cells were treated with 0, 10, or 20 *μ*M of destruxin B for 12 and 24 h. The cells were collected, lyzed, and subjected to western blot with antibodies against PUMA, Bax, Bak, Mcl-1, Bcl-xL, Bcl-2, and *β*-actin, respectively. (b) Upregulation of PUMA but not Mcl-1 RNA expression by destruxin. A549 cells were treated with or without 20 *μ*M destruxin B for 8, 12, and 24 h. The cells were then collected and the total RNA was extracted followed by real-time PCR using PUMA (upper panel) or Mcl-1 (bottom panel) primer set. The actin primer set was also applied as an internal control. The normalized relative expression folds of PUMA between untreated and treated cells were quantified (*P < 0.05 versus control; **P < 0.01 versus control; ***P < 0.001 versus control).

It is well documented that Bcl-2 family molecule plays a central role in mitochondria-mediated apoptosis. To examine the mitochondrial apoptotic events associated with destruxin-B-induced apoptosis, we examined the levels of proapoptotic and antiapoptotic proteins by western blot analysis in whole lysates of A549 cells treated with 0, 10, and 20 *μ*M destruxin for 24 and 48 h. It was found that destruxin B treatment markedly increased levels of the proapoptotic protein PUMA and reduced levels of antiapoptotic proteins Mcl-1 in a concentration- and time-dependent manner (Figure 3(a)). However, the levels of Bax, Bak, Bcl-2, and Bcl-xL were not affected by destruxin. To clarify whether destruxin B regulated the expression of PUMA and Mcl-1 at a transcriptional level, the mRNA expression was analyzed by qPCR. The results showed that addition of destruxin B drastically increased the level of PUMA mRNA, (Figure 3(b), upper panel), strongly indicating that increasing PUMA expression by destruxin B might attribute to transcriptional regulation. In contrast, the mRNA level of Mcl-1 was not affected by destruxin B (Figure 3(b), bottom panel), suggesting that destruxin-mediated Mcl-1 protein level reduction might be regulated by a posttranslational manner. 

### 3.4. Mitochondrial Translocation of Bax Is Important for the Destruxin-Induced Apoptosis

**Figure 4:**
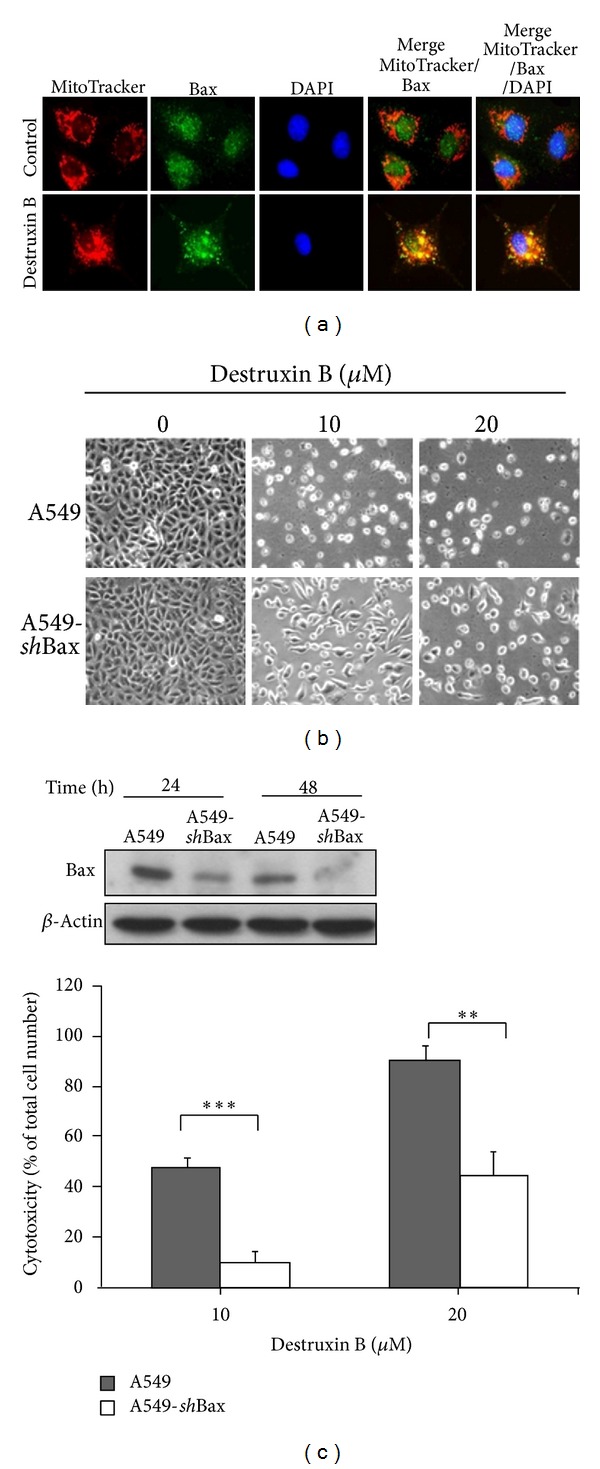
Bax plays a crucial role in destruxin-B-induced apoptosis. (a) A549 cells were untreated or treated with 20 *μ*M destruxin B for 24 h, and the cells were then fixed with 4% paraformaldehyde and subjected to indirect immunofluorescence by using anti-Bax antibody (green color); DAPI (blue color) was used as a nuclear staining, and MitoTracker (red color) was used as a mitochondrial tracker. (b, c) The A549 and A549-*sh*Bax cells were treated with 0, 10, and 20 *μ*M destruxin B for 72 h, (b) the cell morphology was observed under phase-contrast microscopy, and (c) the cytotoxicity of destruxin B was determined. **P < 0.01 versus control; ***P < 0.001 versus control.

It has been reported that during mitochondria-mediated apoptosis, Bax can translocate from cytosol to mitochondrial membrane and form pores to disrupt the permeability of the mitochondrial membrane. We then investigated whether Bax contributed to destruxin-B-mediated mitochondrial apoptosis. Data from immunofluorescence staining showed that Bax exhibited a diffuse distribution throughout the untreated A549 cells (Figure 4(a)). However, administration of destruxin B resulted in a subcellular redistribution of Bax, the immunoreactivity of Bax formed a punctate pattern, and the yellow color in the overlay of Figure 4(a) pointed out the colocalization of Bax with mitochondrial, indicating the translocation of Bax from cytosol to mitochondria upon destruxin treatment. To characterize the role of Bax in destruxin-B-induced apoptosis, we compared the effects of destruxin B on cell viability between parental A549 and A549-*sh*Bax cells (which were stably transfected with two Bax-specific *sh*RNAs). As indicated in Figure 4(b), A549-*sh*Bax cells showed more resistance to the destruxin-B-induced cytotoxicity than parental A549 cells (Figures 4(b) and 4(c)). Approximately 89.9% and 43.9% apoptotic cells were detected in A549 and A549-*sh*Bax cells in response to 20 *μ*M destruxin B treatment, respectively. These results clearly demonstrate that Bax is a major proapoptotic molecule in regulation of apoptosis primed by destruxin B in A549 cells.

### 3.5. Destruxin B Triggers Mitochondria-Mediated Apoptosis in H1299 Cells

**Figure 5:**
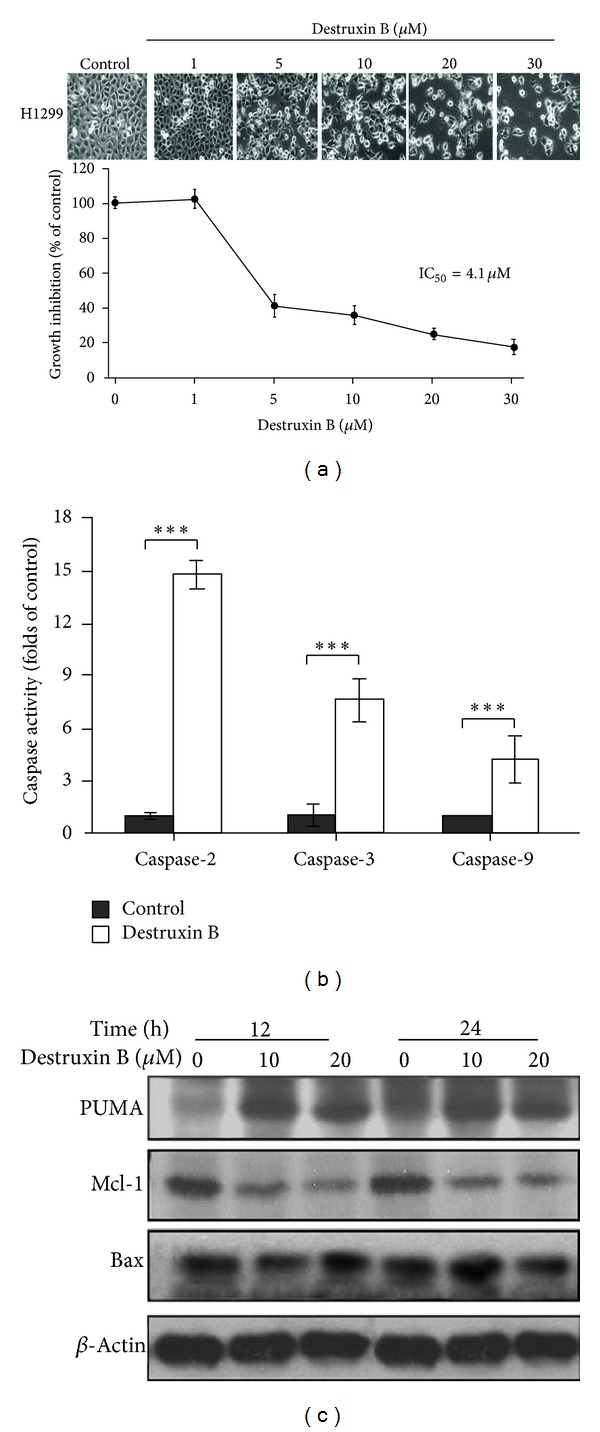
The effects of destruxin B on H1299 cells. (a) Antiproliferative effect. H1299 cells were untreated or treated with 1, 5, 10, 20, and 30 *μ*M of destruxin B for 48 h. The cell number was measured. (b) Activation of caspases. H1299 cells were treated with 20 *μ*M destruxin B for 48 h. The cells were then lyzed and incubated with the fluorescein-conjugated substrates of caspase-2, -3, and -9 and subjected to the microplate reader to detect caspase activity. *P < 0.05 versus control; **P < 0.01 versus control; ***P < 0.001 versus control. (c) H1299 cells were treated with 0, 10, or 20 *μ*M destruxin B for 12 and 24 h. The cells were collected, lyzed, and subjected to western blot with antibodies against PUMA, Mcl-1, and *β*-actin, respectively.

We expanded our study to another human lung cancer cell line, H1299. Destruxin B also inhibited cell proliferation of H1299 cells, with IC_50_ value at 4.1 *μ*M (Figure 5(a)). Consistently, the administration of destruxin B significantly stimulated the activation of caspase-2, -3, and -9 (Figure 5(b)). Moreover, destruxin B increased the level of PUMA while decreased Mcl-1 in H1299 cells (Figure 5(c)). These results indicate that modulation of Bcl-2 family molecules and activation of mitochondria-mediated caspase are required for destruxin-induced apoptosis. 

## 4. Discussion

Lung cancer has been the most common malignancy in the world for several decades that carries poor prognosis. Although substantial progress has been made in developing chemotherapeutic treatments for lung cancer, drug efficacy is often outweighed by undesirable side effects. Nowadays, lung cancer is still the first leading cause of death from cancer, with 1.4 million deaths annually. Thus, it is crucially important to develop better therapeutic strategies for the management of lung cancer. In the recent years, phytochemicals and microbial extracts isolated from various sources have shown significant anticancer activities [30]. It is estimated that approximately 50% of the drugs currently used in the clinic are derived from the natural products or their synthetic analogs, which still continue to provide essential sources of novel discovery leads. Cyclodepsipeptides, the secondary metabolites of fungi and plants [31], show a broad spectrum of biological activity, including immunosuppressant, antibiotic, antifungal, anti-inflammatory, and anticancer effects [31]. Some of these natural products and (semi-)synthetic derivatives have already been evaluated in clinical trials [31]. Destruxin B, one of the cyclodepsipeptides, is isolated and purified from entomopathogenic fungi. Previous studies demonstrate that destruxin B has insecticidal and antiviral effects [24]. Additionally, our studies and other studies demonstrate that destruxin B has antitumor effect in leukemia cells [32], hepatocellular carcinoma cells [25], and colorectal cancer [33]. This study firstly reported that destruxin B inhibits cell proliferation and induces apoptotic cell death in human nonsmall cell lung cancer cell lines, A549 and H1299. Moreover, destruxin-B-triggered apoptosis is through a Bax-dependent mitochondria-mediated caspase activation pathway.

Our paper is the first report to conduct a mechanistic study to investigate how destruxin B induces apoptosis. It is well documented that apoptosis is activated mainly through two pathways: the mitochondria-dependent pathway (intrinsic pathway) and the death receptor pathway (extrinsic pathway). Bcl-2 family proteins play a key role in the regulation of mitochondria-dependent apoptosis [34]. The relative equilibrium of various anti- and proapoptotic Bcl-2 family members are a critical determinant of cell apoptosis. Bax is mainly localized in the cytosol of healthy cells as a soluble monomeric protein in an apoptotically inactive status [35]. In response to apoptotic cues, Bax undergoes conformational changes and then translocates to the mitochondrial outer membrane and forms large oligomeric complexes, thereby triggering mitochondrial dysfunction and caspase activation to bring about apoptosis. Mcl-1 can directly bind with Bax and prevent apoptotic activation of Bax [36]. In this study, we found that treatment with destruxin B resulted in decrease of Mcl-1, increase of PUMA, and induction of Bax mitochondrial translocation. Moreover, silencing Bax expression by *sh*RNA effectively attenuated destruxin-B-triggered apoptosis. This suggests that Bax plays a more crucial role in conducting destruxin-B-mediated cytotoxicity. It is known that PUMA is a general sensor of apoptotic stimuli and a promising drug target for cancer therapy [37]. Biochemical studies indicate that PUMA induces apoptosis by activating the proapoptotic protein Bax through its interaction with antiapoptotic Bcl-2 family members, including Bcl-2, Bcl-xL, Mcl-1, Bcl-w, and A1 [38]. The interactions of PUMA with antiapoptotic proteins cause displacement of Bax, resulting in activation of the proapoptotic activity of Bax. PUMA overexpression leads to a conformational change, multimerization, and mitochondrial translocation of Bax, which induces apoptotic cell death [37]. Based on these data, we propose that one of the mechanisms by which destruxin-B-induced apoptosis was by overexpression of PUMA and decrease of Mcl-1 resulting in disruption of Mcl-1/Bax complexes, triggering Bax oligomerization and translocation to mitochondria in human nonsmall cell lung cancer cells.

The apoptosis process is mediated by sequential activation of caspases. Caspases, a family of cysteine proteases, is one of the biomarkers of apoptosis [39]. Once activated, caspases can cleave hundreds of cellular proteins resulting in the hallmark morphological characteristics of apoptosis. Accumulating evidence indicates that activation of caspase cascade plays a key role in the induction of apoptosis for many cancer therapeutic drugs in many types of cancer cells [39]. In particular, activation of caspase-3 plays a central role in the execution of apoptosis. Activation of caspase-3 requires the activation of initiator caspases, such as caspase-8 or caspase-9, in response to proapoptotic signals. It is well known that caspase-9 is the major initiator caspase of mitochondria-mediated apoptosis while caspase-8 is the key initiator caspase of death receptor-triggered apoptosis. Our current study clearly demonstrated that treatment of A549 cells with destruxin B enhanced the activation of caspase-9 and -3. Reduction of caspase cascade with specific inhibitors effectively blocked destruxin-B-induced apoptosis. These results indicated that destruxin-B-induced apoptosis was through a mitochondria-dependent caspase-9/-3-activated response. Interestingly, this study also showed that caspase-2 was activated in A549 and H1299 cells upon destruxin B administration. Caspase-2 is considered as both initiator and executor caspase in apoptosis [40]. Caspase-2 has been shown to act upstream of the mitochondria and is involved in Bid cleavage and Bax translocation, which results in cytochrome c release during genotoxic stress [41]. Therefore, we proposed that the activation of caspase-2 by destruxin B might be one of the important regulators in inducing Bax translocation and subsequent mitochondrial dysfunction, finally leading to apoptosis. 

**Figure 6:**
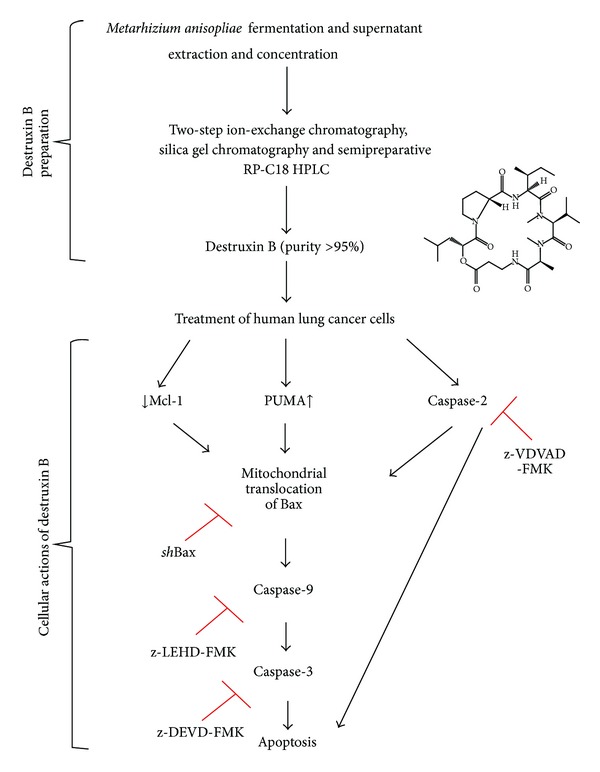
Schematic representation of destruxin B preparation and mechanistic illustration of destruxin-B-induced apoptosis. Destruxin B isolated from *Metarhizium anisopliae *induces apoptosis in human nonsmall cell lung cancer cells by activating a mitochondrial-dependent caspase activation signaling pathway.

In conclusion, the present study supports that destruxin B isolated and purified from *Metarhizium anisopliae* F061 var. Anisopliae induces the intrinsic apoptotic pathway in human nonsmall cell lung cancer A549 cells, and a signaling cascade is proposed in Figure 6. Destruxin-B-mediated upregulation of PUMA and downregulation of Mcl-1 may facilitate the mitochondrial translocation of Bax, subsequently inducing caspase-2, -9, and -3 activation and apoptosis. Importantly, inhibition of Bax expression by *sh*RNA significantly diminishes destruxin-B-induced cytotoxicity, suggesting that Bax-dependent mitochondrial pathway contributes to destruxin B activity in A549 cells. Because some of these results have been confirmed in H1299 cells, we hypothesize that destruxin B may activate some common signaling events in different human nonsmall cell lung cancer cell types.
